# Antibiotic use in children with suspected CNS infection in the molecular diagnostic era: a cohort study of 1198 children

**DOI:** 10.1093/jacamr/dlag199

**Published:** 2026-09-02

**Authors:** Yannik Vollmuth, Katrin Baalmann, Jana Beer, Benjamin Soric, Uta Behrends, Melanie Meyer-Bühn, Christoph Klein, Johannes Hübner, Gerhard Dobler, Tilmann Schober

**Affiliations:** Department of Pediatrics, Dr. von Hauner Children’s Hospital, LMU University Hospital, LMU Medizin, Ludwig-Maximilians-Universität München, Munich, Germany; German Center for Infection Research (DZIF), Partner Site Munich, Munich, Germany; Department of Pediatrics, Dr. von Hauner Children’s Hospital, LMU University Hospital, LMU Medizin, Ludwig-Maximilians-Universität München, Munich, Germany; Department of Pediatrics, Dr. von Hauner Children’s Hospital, LMU University Hospital, LMU Medizin, Ludwig-Maximilians-Universität München, Munich, Germany; Department of Pediatrics, Dr. von Hauner Children’s Hospital, LMU University Hospital, LMU Medizin, Ludwig-Maximilians-Universität München, Munich, Germany; German Center for Infection Research (DZIF), Partner Site Munich, Munich, Germany; Children's Hospital, School of Medicine, Technical University of Munich, Munich, Germany; Department of Pediatrics, Dr. von Hauner Children’s Hospital, LMU University Hospital, LMU Medizin, Ludwig-Maximilians-Universität München, Munich, Germany; Department of Pediatrics, Dr. von Hauner Children’s Hospital, LMU University Hospital, LMU Medizin, Ludwig-Maximilians-Universität München, Munich, Germany; German Center for Infection Research (DZIF), Partner Site Munich, Munich, Germany; German Center for Child and Adolescent Health (DZKJ), Göttingen, Germany; Department of Pediatrics, Dr. von Hauner Children’s Hospital, LMU University Hospital, LMU Medizin, Ludwig-Maximilians-Universität München, Munich, Germany; German Center for Infection Research (DZIF), Partner Site Munich, Munich, Germany; National TBEV Consultant Laboratory, Bundeswehr Institute of Microbiology, Munich, Germany; Department of Tropical Medicine and Infectious Diseases, LMU Medizin, Ludwig-Maximilians-Universität München, Munich, Germany; Department of Pediatrics, Dr. von Hauner Children’s Hospital, LMU University Hospital, LMU Medizin, Ludwig-Maximilians-Universität München, Munich, Germany

## Abstract

**Objectives:**

Central nervous system (CNS) infections in children require rapid diagnostic evaluation and prompt empiric antimicrobial therapy. However, clinical presentation is often non-specific, leading to frequent use of broad-spectrum antibiotics while awaiting cerebrospinal fluid (CSF) results. We evaluated antibiotic treatment modifications during the diagnostic work-up and quantified changes in antibiotic spectrum coverage (ASC) before and after lumbar puncture (LP).

**Patients and methods:**

We conducted a retrospective single-centre study including children <18 years who underwent LP for suspected CNS infection between January 2016 and December 2024. CSF was analysed using the BioFire^®^ FilmArray^®^ Meningitis/Encephalitis (ME) Panel. Antibiotic treatment modifications after LP-related diagnostic information became available were assessed descriptively. Paired ASC scores before and after LP were compared using the Wilcoxon signed-rank test among treated children.

**Results:**

Among 1198 children undergoing LP, 730 (60.9%) received antibiotic therapy. Antibiotic use was highest in neonates (227/246; 92.3%) and infants aged 29 days to <6 months (118/140; 84.3%) and lowest in adolescents (106/329; 32.2%). Treatment was modified after LP-related diagnostic information became available in 412/1198 children (34.4%). Among treated children, 313 (42.9%) underwent de-escalation, 99 (13.6%) escalation and 318 (43.6%) had no change. Overall, mean ASC decreased from 8.5 before LP to 6.6 afterwards (ΔASC −1.9; *P* < 0.001).

**Conclusions:**

LP-related diagnostic information was associated with frequent antibiotic treatment modification and reduced antibiotic spectrum coverage. These findings support integrated CSF diagnostics, including multiplex polymerase chain reaction, as a component of antimicrobial stewardship in children with suspected CNS infection.

## Introduction

Infections of the central nervous system (CNS), particularly bacterial meningitis, remain life-threatening emergencies associated with significant morbidity and mortality despite improved prevention and treatment strategies.^1^ In children, CNS infections such as meningitis and encephalitis therefore require prompt diagnostic evaluation and initiation of empiric antimicrobial therapy. However, clinical presentation in paediatric patients is often non-specific, and broad-spectrum antimicrobial treatment is frequently initiated while awaiting microbiological results from cerebrospinal fluid (CSF) analysis.^2^ In the era of conjugate vaccination, bacterial meningitis has become increasingly rare, viral pathogens predominate, and many cases remain without an identified aetiology despite extensive diagnostic work-up.^3–5^ Our previously published epidemiological analysis of the same cohort, which demonstrated that definite bacterial meningitis was rare and non-bacterial presentations predominated, provides the clinical context for the present analysis of antibiotic exposure and treatment modification.^5^

Rapid molecular diagnostics, particularly multiplex polymerase chain reaction (PCR) panels such as the FilmArray meningitis/encephalitis (ME) panel, enable simultaneous detection of multiple bacterial and viral pathogens directly from CSF within a short turnaround time and have substantially improved diagnostic yield.^2,6^ While these technologies may support antimicrobial stewardship by facilitating earlier optimization of antimicrobial therapy, their impact on antibiotic prescribing patterns has been heterogeneous across studies.^6,7^ A 2022 meta-analysis reported that, in paediatric studies, the use of the multiplex ME panel was associated with a non-significant reduction in hospital length of stay (1.1 day) and significant reductions in both acyclovir (1.7 days) and antibiotic therapy (1.9 days).^6,8–12^ These studies have assessed the implementation of multiplex PCR diagnostics. The impact on antibiotic prescribing during the diagnostic process remains unclear.

While days of therapy remains the standard metric for measuring antimicrobial utilization, it does not account for differences in antimicrobial spectrum; therefore, spectrum-based scoring systems have been developed to better quantify antibiotic exposure and support antimicrobial stewardship.^13^ To our knowledge, spectrum-based metrics have not been studied to evaluate antimicrobial therapy in children with suspected CNS infections.

Therefore, the aim of this study was to evaluate changes in antimicrobial therapy during the diagnostic work-up of children with suspected CNS infection, including antibiotic initiation, discontinuation, escalation, de-escalation and switching, and to complement these direct treatment modifications by quantifying changes in antibiotic spectrum coverage before and after lumbar puncture.

## Patients and methods

### Ethics

The study was approved by the Ethics Committee of the LMU Munich (project number 24-1003) and conducted in accordance with the Declaration of Helsinki. The Strengthening the Reporting of Observational studies in Epidemiology (STROBE) guidelines were followed [see corresponding checklist, Supplemental Material (available as Supplementary data at *JAC-AMR* Online)].^14^

### Study design and patient population

This retrospective, single-centre study was conducted at Dr. von Hauner Children’s Hospital, the tertiary-care paediatric hospital of Ludwig-Maximilians-Universität (LMU) Munich, Germany. Children and adolescents <18 years of age were eligible if they underwent lumbar puncture (LP) for suspected CNS infection between January 2016 and December 2024 and had CSF multiplex PCR testing performed. Throughout the study period, CSF multiplex PCR was part of routine clinical care for children undergoing LP for suspected CNS infection. Testing was performed using the BioFire^®^ FilmArray^®^ ME Panel (BioMérieux, Marcy-l’Étoile, France).

Patients were excluded if they had a ventricular shunt, had undergone recent neurosurgery or if LP was unsuccessful with no CSF available for analysis. For patients with multiple hospital admissions, only the first admission with LP was included.

The epidemiology, clinical phenotypes and diagnostic findings of this cohort have been reported previously.^5^ The present study uses the same cohort but addresses a distinct research question examining antibiotic exposure, treatment modifications and changes in antibiotic spectrum coverage; antimicrobial and antiviral treatment data were not analysed in the previous publication.

### Variables of interest and study definitions

Data were retrospectively extracted from electronic medical records. Demographic, clinical, laboratory and additional variables are summarized in Table 2 and described in detail in the online Supplementary Methods.

Meningitis was classified according to predefined criteria adapted from the UK-ChiMES network, based on clinical features, CSF findings and microbiological results.^4^

Encephalitis was classified according to the International Encephalitis Consortium consensus criteria.^15^ The major criterion was altered mental status, lethargy or personality change lasting ≥24 h without another cause. Minor criteria were fever ≥38°C within 72 h, unexplained seizures, new focal neurological deficits, CSF white blood cell count ≥5 cells/mm^3^, suggestive neuroimaging or compatible electroencephalographic abnormalities. Cases were classified as possible or probable encephalitis according to the number of minor criteria fulfilled (Figure S1; Table S2).^15^ Cases of probable encephalitis, with additional microbiological confirmation of a causative pathogen, were defined as confirmed encephalitis. The control group was defined as patients without CSF pleocytosis, without pathogen detection in CSF multiplex PCR and without a diagnosis of possible or probable encephalitis (Table S2).

For the analysis, three antibiotic exposure groups were defined. First, the ‘any antibiotic therapy group’ included all patients who received antibiotics, regardless of regimen. Second, the ‘meningitis-directed antibiotic therapy group’ included patients treated with established empiric meningitis therapy (ceftriaxone, cefotaxime, ceftazidime or meropenem), irrespective of timing in relation to LP. Third, the antibiotic change after LP results group included patients whose antimicrobial therapy was modified after LP results became available, including initiation, discontinuation, escalation, de-escalation or switching of antibiotics.

Antibiotic exposure was quantified using the antibiotic spectrum coverage (ASC) score, which assigns numeric values to antimicrobial agents according to the breadth of pathogens covered. Higher scores indicate broader-spectrum therapy.^13,16,17^ Antibiotic modification was defined as any change in antimicrobial therapy after LP, including initiation, discontinuation, escalation, de-escalation or switching of antibiotics. ASC was calculated for antibiotic therapy before LP (ASC pre-LP) and after LP (ASC post-LP). The change in antibiotic spectrum (ΔASC) was defined as the difference between post- and pre-LP ASC values and was compared using the Wilcoxon signed-rank test. For descriptive analyses, ASC values were additionally categorized to facilitate interpretation as none (ASC = 0), moderate spectrum (ASC 1–9) and broad spectrum (ASC >10), as presented in Tables S7 and S8.

### Data analysis

Data collection and preparation of tables and figures were performed using Microsoft Excel, Word and PowerPoint (Microsoft Corp., Redmond, WA, USA) and BioRender (Toronto, ON, Canada). Sankey diagrams were generated in Python (Python Software Foundation, Wilmington, DE, USA), and statistical analyses were conducted in SPSS (IBM Corp., Armonk, NY, USA). Categorical variables are reported as frequencies and percentages and continuous variables as medians with interquartile ranges. Two-sided *P* values <0.05 were considered significant. Pre- and post-LP ASC values were compared using the Wilcoxon signed-rank test. Missing data were analysed using an available-case approach without imputation. Antibiotic exposure variables were complete for the full cohort, while paired ASC analyses were limited to the 730 children receiving antibiotics.

## Results

**Figure 1.**
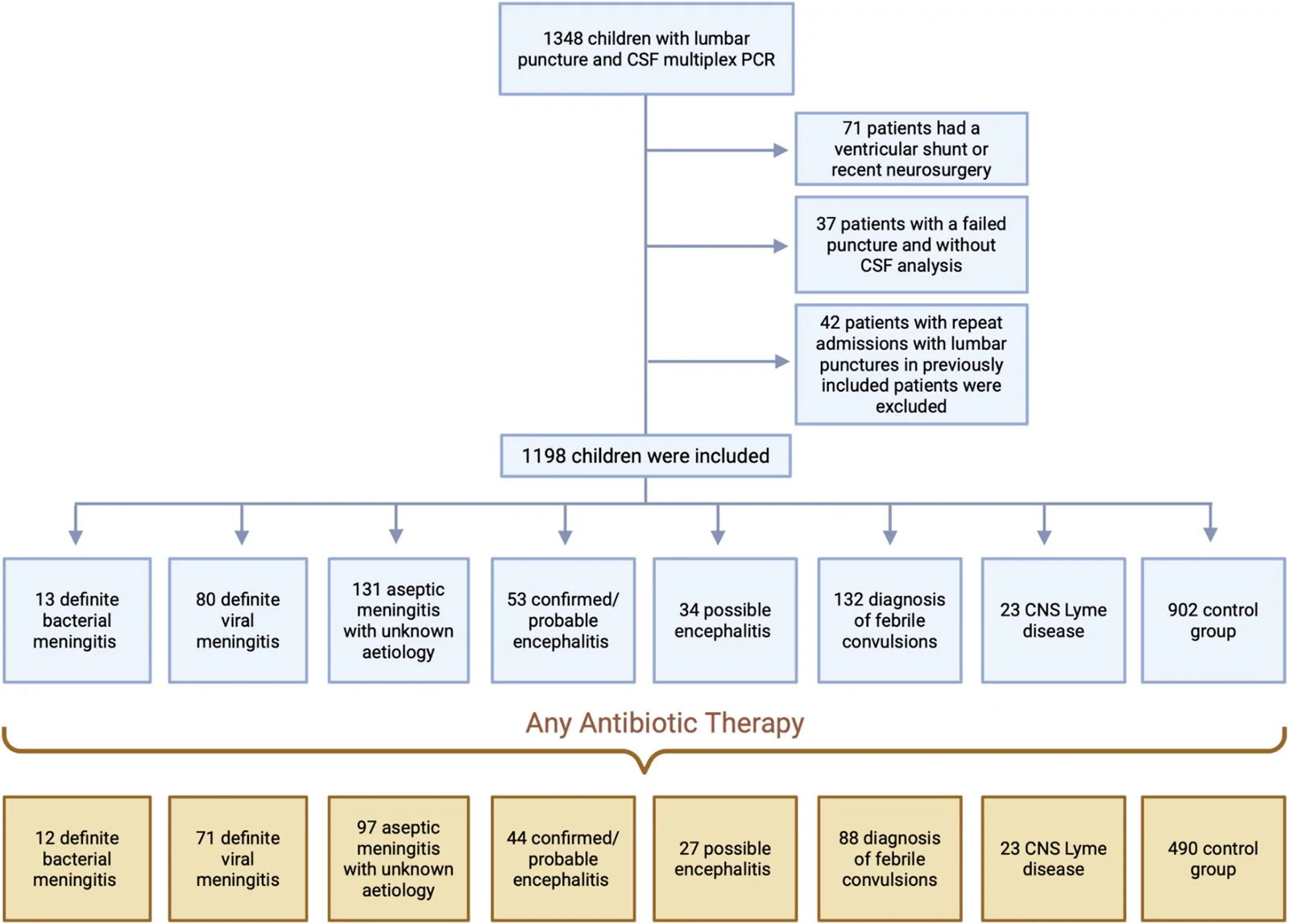
Flowchart of hospitalized children <18 years of age with suspected CNS infection between January 2016 and December 2024.

A total of 1348 children undergoing LP with multiplex PCR were initially assessed for eligibility (Figure 1). After excluding 71 patients with ventricular shunt or recent neurosurgery, 37 patients without CSF analysis and 42 repeat admissions, 1198 children were included in the final analysis.

### Antibiotic treatment according to baseline characteristics

Overall, 730/1198 (60.9%) children received antibiotic therapy at any time point, 622/1198 (51.9%) received meningitis-directed antibiotic therapy for suspected meningitis, and in 412/1198 (34.4%) children, antibiotic therapy was changed after availability of LP results (Table 1).

**Table 1.** Antibiotic treatment according to baseline characteristics

	Any antibiotic therapy		Meningitis-directed antibiotic therapy		Antibiotic change after LP results		Total^a^
	Absolute numbers [proportion of any antibiotic therapy (%)]	Proportion of total	Absolute numbers [proportion of antibiotic meningitis therapy (%)]	Proportion of total	Absolute numbers [proportion of antibiotic change after LP (%)]	Proportion of total	
n	730	60.9%	622	51.9%	412	34.4%	1198
Sex, n							
Female	403 (55.1)	73.5%	270 (43.4)	49.3%	188 (45.6)	34.3%	548 (45.7)
Male	327 (44.8)	50.3%	352 (56.6)	54.2%	224 (54.4)	34.4%	650 (54.3)
Age groups, n							
0–28 days	227 (31.1)	92.3%	200 (32.2)	81.3%	130 (31.6)	52.8%	246 (20.5)
29 days to <6 months	118 (16.2)	84.3%	106 (17.0)	75.7%	78 (18.9)	55.7%	140 (11.7)
6–12 months	40 (5.5)	59.7%	34 (5.5)	50.7%	23 (5.6)	34.3%	67 (5.6)
1–4 years	138 (18.9)	57.3%	120 (19.3)	49.8%	82 (19.9)	34.0%	241 (20.1)
5–9 years	101 (13.8)	57.7%	82 (13.2)	46.9%	43 (10.4)	24.6%	175 (14.6)
10–17 years	106 (14.5)	32.2%	80 (12.9)	24.3%	56 (13.6)	17.0%	329 (27.5)
Past medical history, n							
Immunocompromised	52 (7.1)	71.2%	30 (4.8)	41.1%	22 (5.3)	30.1%	73 (6.1)
Malignancy	30 (4.1)	66.7%	17 (2.7)	37.8%	13 (3.2)	28.9%	45 (3.8)
Previous neurological condition	49 (6.7)	44.5%	37 (5.9)	33.6%	23 (5.6)	20.9%	110 (9.2)
Vaccines up to date, n							
Yes	563 (77.1)	66%	488 (78.5)	57.2%	330 (80.1)	38.7%	853 (71.2)
No	41 (5.6)	53.2%	38 (6.1)	49.4%	21 (5.1)	27.3%	77 (6.4)
Not reported	126 (17.3)	47.0%	96 (15.4)	35.8%	61 (14.8)	22.8%	268 (22.4)
History, n							
Symptom duration (days) before hospital presentation (median, IQR)	0.0 (0.0–2.0)		0.0 (0.0–2.0)		0.0 (0.0–1.0)		1 (0.0–3.0)

^a^The total represents the overall study population and is not the sum of the individual diagnostic groups.

Antibiotic use demonstrated a strong age-related gradient. The highest proportion of antibiotic therapy was seen in neonates (227/246; 92.3%). Similarly, high proportions were observed among infants aged 29 days to <6 months (118/140; 84.3%). In contrast, the proportion of children receiving antibiotics decreased markedly with increasing age. In children aged 1–4 years and 5–9 years, the proportion was similar (138/241; 57.3%) and (101/175; 57.7%), respectively. In adolescents aged 10–17 years, antibiotic therapy was considerably less frequent (106/329; 32.2%) (Table 1). A similar age-related gradient was observed for meningitis-directed antibiotic therapy for suspected meningitis; in this subgroup, limited to patients receiving established empiric meningitis treatment, proportions decreased from 81.3% in neonates to 24.3% in adolescents aged 10–17 years (Table 1).

Antibiotic modification after LP results also showed an age-dependent pattern. Changes in therapy occurred most frequently in younger children: 130/246 (52.8%) in neonates and 78/140 (55.7%) in infants aged 29 days to <6 months. In children aged 6–12 months and 1–4 years, antibiotic changes were observed in 23/67 (34.3%) and 82/241 (34.0%), respectively. The proportion decreased further to 43/175 (24.6%) in children aged 5–9 years and to 56/329 (17.0%) in adolescents aged 10–17 years (Table 1).

### Antibiotic treatment according to clinical characteristics and outcomes

All children with hospital-acquired suspected infection (7/7; 100.0%) received antibiotic therapy. In contrast, among community-acquired suspected infections, antibiotic therapy was administered in 723 of 1191 children (723/1191; 60.8%) (Table 2). Several symptoms were strongly associated with antibiotic therapy. Antibiotics were most frequently administered in children presenting with bulging fontanelle (9/10; 90.0%), neck stiffness (66/78; 84.6%), fever (396/495; 80.0%), rash (79/99; 79.8%), irritability (153/193; 79.3%) and altered level of consciousness >24 h (104/136; 76.5%). A similar pattern was observed among children who received meningitis-directed antibiotic therapy (Table 2).

**Table 2.** Antibiotic treatment according to clinical characteristics and outcomes

	Any antibiotic therapy		Meningitis-directed antibiotic therapy		Antibiotic change after LP results		Total^a^
	Absolute numbers [proportion of any antibiotic therapy (%)]	Proportion of total	Absolute numbers [proportion of antibiotic meningitis therapy (%)]	Proportion of total	Absolute numbers [proportion of antibiotic change after LP (%)]	Proportion of total	
n	730	60.9%	622	51.9%	412	34.4%	1198
Hospital-acquired, n	7 (1.0)	100.0%	4 (0.6)	57.1%	1 (0.2)	14.3%	7 (0.6)
Community-acquired, n	723 (99.0)	60.8%	618 (99.4)	51.9%	411 (99.7)	34.5%	1191 (99.4)
Symptoms, n							
Fever	396 (54.2)	80.0%	339 (54.5)	68.5%	221 (53.6)	44.6%	495 (41.3)
Headache	93 (12.7)	41.3%	77 (12.4)	34.2%	44 (10.7)	19.6%	225 (18.8)
Neck stiffness	66 (9.0)	84.6%	63 (10.1)	80.8%	38 (9.2)	48.7%	78 (6.5)
Irritability	153 (21.0)	79.3%	143 (23.0)	74.1%	100 (24.3)	51.8%	193 (16.1)
Rash	79 (10.8)	79.8%	64 (10.3)	64.6%	44 (10.7)	44.4%	99 (8.3)
Vomiting	151 (20.7)	65.7%	134 (21.5)	58.3%	81 (19.7)	35.2%	230 (19.2)
Bulging fontanelle	9 (1.2)	90.0%	9 (1.4)	90.0%	5 (1.2)	50.0%	10 (0.8)
Altered level of consciousness >24 h	104 (14.2)	76.5%	91 (14.6)	66.9%	53 (12.9)	39.0%	136 (11.4)
Personality change >24 h	35 (4.8)	47.9%	29 (4.7)	39.7%	20 (4.9)	27.4%	73 (6.1)
Seizure <72 h before or during hospital stay	205 (28.1)	62.5%	176 (28.3)	53.7%	119 (28.9)	36.3%	328 (27.4)
New-onset focal neurological deficit	150 (20.5)	46.6%	129 (20.7)	40.1%	79 (19.2)	24.5%	322 (26.9)
Outcome, n							
Length of hospital stay (median, IQR)^b^	6.0 (4.0–9.3)		6.0 (4.0–9.0)		5.0 (4.0–8.0)		4.0 (3.0–7.0)
ICU stay	267 (36.6)	77.8%	221 (35.5)	64.4%	123 (29.9)	35.9%	343 (28.6)
Death	5 (0.7)	55.6%	3 (0.5)	33.3%	2 (0.5)	22.2%	9 (0.8)

^a^The total represents the overall study population and is not the sum of the individual diagnostic groups

^b^Length of hospital stay refers to the duration of hospitalization and not to the duration of antibacterial or antiviral therapy.

Antibiotic modification after LP results was also more frequent in children presenting with these symptoms. Changes in therapy were observed in children with irritability (100/193; 51.8%), bulging fontanelle (5/10; 50.0%), neck stiffness (38/78; 48.7%), fever (221/495; 44.6%), rash (44/99; 44.4%) and altered level of consciousness >24 h (53/136; 39.0%) (Table 2). Other symptoms were associated with lower proportions of antibiotic therapy, namely personality change >24 h (35/73; 47.9%), new-onset focal neurological deficit (150/322; 46.6%) and headache (93/225; 41.3%). Similar trends were observed for meningitis-directed antibiotic therapy and antibiotic modification after LP results (Table 2).

Patients receiving antibiotic therapy had a longer hospital stay compared with the overall cohort. The median length of stay was 6.0 days (IQR 4.0–9.3) in children receiving any antibiotic therapy and 6.0 days (IQR 4.0–9.0) in those receiving meningitis-directed antibiotic therapy, compared with 4.0 days (IQR 3.0–7.0) in the overall study population. A similar trend was observed among children with antibiotic modification after LP results, with a median length of stay of 5.0 days (IQR 4.0–8.0) (Table 2).

### Impact of diagnostic classification

Antibiotic therapy varied markedly across diagnostic groups. The highest treatment rates were observed in children with CNS Lyme disease (23/23; 100.0%), definite bacterial meningitis (12/13; 92.3%) and definite viral meningitis (71/80; 88.8%), followed by confirmed/probable encephalitis (44/53; 83.0%), possible encephalitis (27/34; 79.4%) and aseptic meningitis of unknown aetiology (97/131; 74.0%). The one patient with definite bacterial meningitis who did not receive antibiotic therapy fulfilled the predefined study criteria for bacterial meningitis; however, the treating physician considered bacterial meningitis clinically unlikely. Lower rates were seen in children with febrile convulsions (88/132; 66.7%) and in the control group (490/902; 54.3%). Similar patterns were observed for meningitis-directed antibiotic therapy, with proportions of 23/23 (100.0%) in Lyme disease, 12/13 (92.3%) in definite bacterial meningitis, 60/80 (75.0%) in definite viral meningitis, 40/53 (75.5%) in confirmed/probable encephalitis, 23/34 (67.6%) in possible encephalitis, 81/131 (61.8%) in aseptic meningitis, 77/132 (58.3%) in febrile convulsions and 414/902 (45.9%) in the control group (Table S4).

Antibiotic modification after lumbar puncture also differed across groups, with the highest proportions in definite bacterial meningitis (9/13; 69.2%) and definite viral meningitis (47/80; 58.8%), followed by Lyme disease (12/23; 52.2%), possible encephalitis (15/34; 44.1%), febrile convulsions (54/132; 40.9%) and confirmed/probable encephalitis (21/53; 39.6%), whereas lower proportions were observed in aseptic meningitis (47/131; 35.9%) and the control group (273/902; 30.3%) (Table S4).

Among the 71 children with definite viral meningitis who received antibiotic therapy, 38 (53.5%) underwent de-escalation, 24 (33.8%) had no change in therapy and 9 (12.7%) underwent escalation (Table S6).

### Impact of COVID-19 pandemic

As the study period from January 2016 to December 2024 encompassed the pre-pandemic, pandemic and post-pandemic periods, an exploratory period-stratified analysis was performed. The monthly number of children undergoing lumbar puncture for suspected CNS infection remained unchanged before and during the pandemic (9.6 and 9.7 children per month, respectively) but increased to 17.4 children per month post-pandemic. The proportion of children receiving any antibiotic therapy differed significantly across the three periods (pre-pandemic: 67.6%; pandemic: 56.6%; post-pandemic: 56.2%; *P* < 0.001). Similar differences were observed for meningitis-directed antibiotic therapy (58.6%, 51.1% and 43.5%, respectively; *P* < 0.001) and for documented treatment changes after LP-related results (44.9%, 23.2% and 31.4%, respectively; *P* < 0.001).

### Antibiotic modification after lumbar puncture

**Figure 2.**
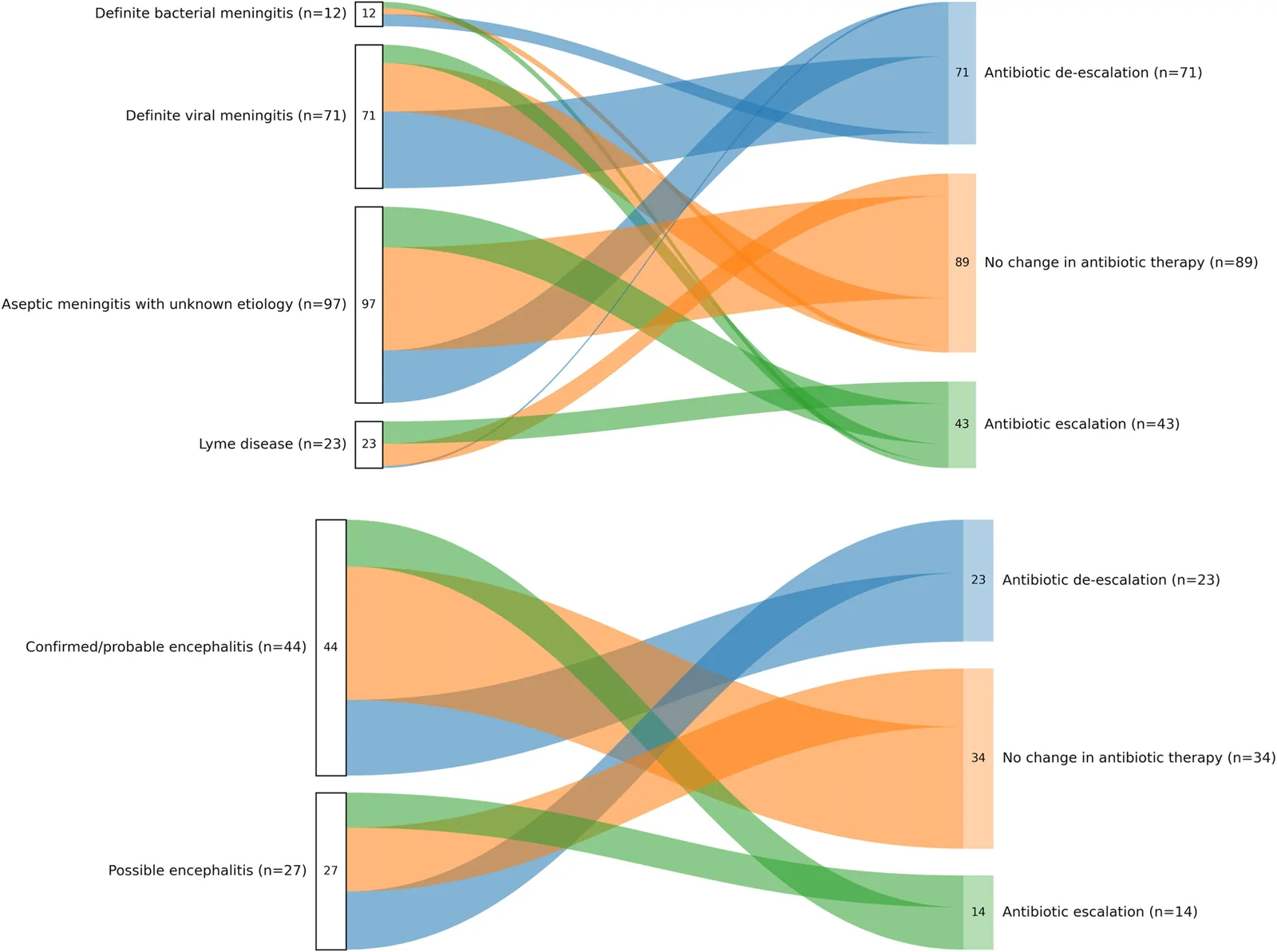
Antibiotic therapy changes after lumbar puncture in meningitis- and encephalitis-related diagnostic groups. *Diagnostic categories may overlap and therefore do not sum to the total cohort.

Following the availability of LP-related diagnostic information, including routine CSF parameters and multiplex PCR results, antibiotic therapy was modified in 412 of 1198 children (34.4%). Among the 730 children who received antibiotic therapy, 313 (42.9%) underwent de-escalation, 99 (13.6%) underwent escalation and 318 (43.6%) had no change in therapy (Figure 2; Table S6). Thus, antibiotic de-escalation occurred more than three times as frequently as escalation.

**Figure 3.**
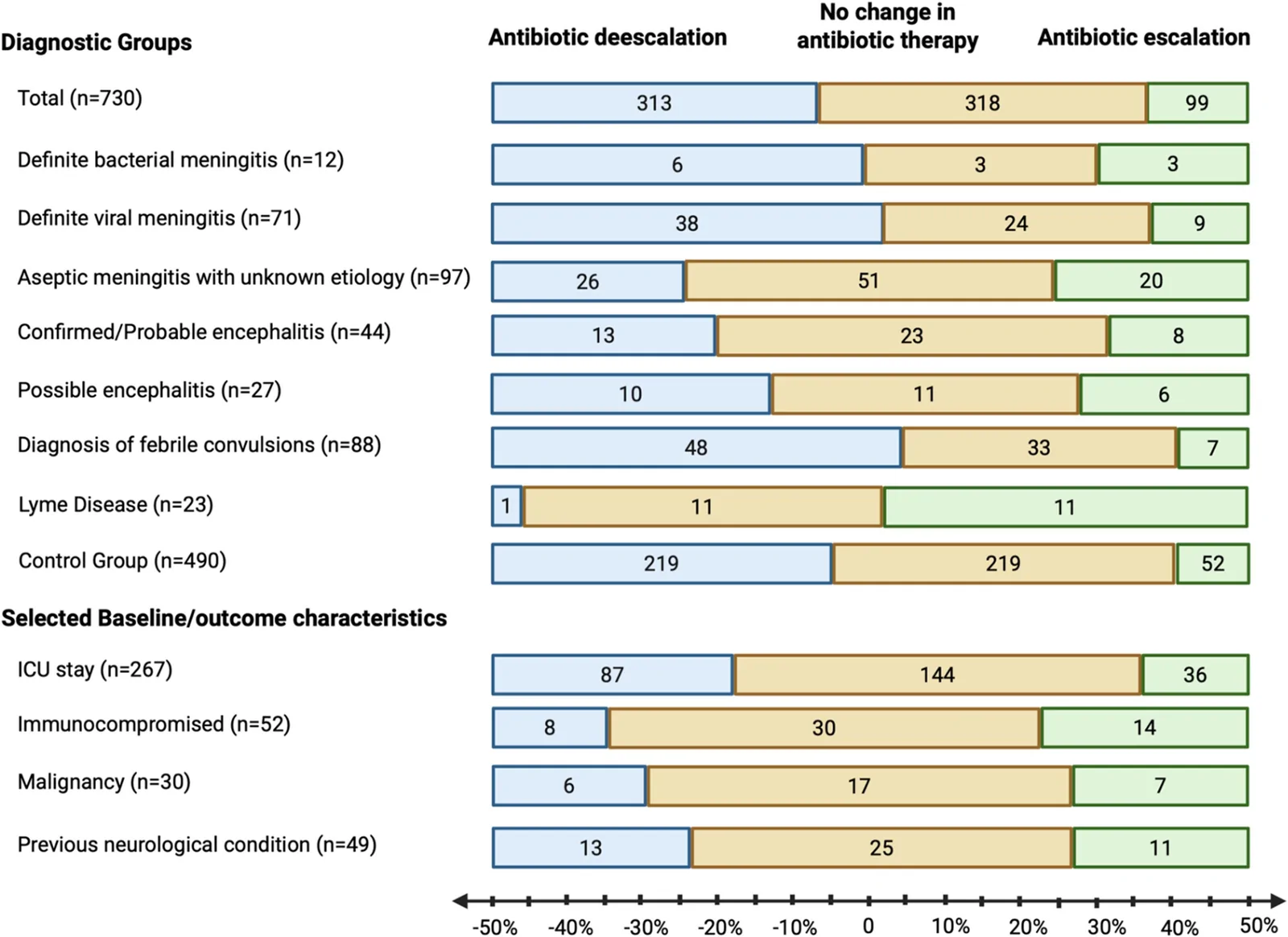
Antibiotic therapy changes after LP by diagnostic classification.

Across the entire cohort receiving antibiotic therapy, the mean ASC score decreased from 8.5 (±3.8) before LP to 6.6 (±4.5) after LP, corresponding to a mean change of −1.9 (±4.6). In the overall group of 730 children who received antibiotic therapy, this reduction in antibiotic spectrum was statistically significant (Wilcoxon signed-rank test: Z = −10.874; *P* < 0.001) (Figure 3; Table 3; Table S5).

**Table 3.** ASC before and after LP by diagnostic classification

	ASC pre-LP (mean ± SD)	ASC post-LP (mean ± SD)	ΔASC (mean ± SD)
Total	8.5 (±3.8)	6.6 (±4.5)	−1.9 (±4.6)
**Diagnostic groups**			
Definite bacterial meningitis	8.8 (±4.8)	7.3 (±4.2)	−1.5 (±5.6)
Definite viral meningitis	9.0 (±4.1)	5.9 (±5.0)	−3.1 (±5.7)
Aseptic meningitis with unknown etiology	8.2 (±4.8)	7.8 (±4.1)	−0.4 (±4.7)
Confirmed/probable encephalitis	7.9 (±4.4)	6.8 (±4.6)	−1.1 (±4.5)
Possible encephalitis	8.6 (±3.9)	8.3 (±7.1)	−0.3 (±5.8)
Diagnosis of febrile convulsions	5.8 (±1.8)	3.1 (±3.6)	−2.7 (±4.6)
Lyme disease	4.5 (±3.4)	6.2 (±0.4)	1.7 (±3.2)
Control group	8.7 (±3.6)	6.6 (±4.2)	−2.2 (±4.2)
**Selected baseline and outcome characteristics**			
ICU stay	9.5 (±3.8)	8.3 (±4.9)	−1.1 (±4.5)
Immunocompromised	10.1 (±5.3)	10.5 (± 4.6)	0.5 (±5.0)
Malignancy	10.9 (±4.8)	10.4 (±4.7)	−0.4 (±4.7)
Previous neurological condition	8.1 (±3.8)	8.2 (±5.4)	0.1 (±5.6)

In the descriptive subgroup analysis, the largest mean reduction in ASC was observed in children with definite viral meningitis, where the mean ASC decreased from 9.0 (±4.1) before LP to 5.9 (±5.0) after LP (ΔASC −3.1 ± 5.7). No subgroup-specific inferential tests were performed. Relevant reductions were also observed in the control group (8.7 ± 3.6 to 6.6 ± 4.2; ΔASC −2.2 ± 4.2) and in children with febrile convulsions (5.8 ± 1.8 to 3.1 ± 3.6; ΔASC −1.8 ± 4.6) (Table 3). More modest reductions were seen in children with definite bacterial meningitis (8.8 ± 4.8 to 7.3 ± 4.2; ΔASC −1.5 ± 5.6), confirmed or probable encephalitis (7.9 ± 4.4 to 6.8 ± 4.6; ΔASC −1.1 ± 4.5) and aseptic meningitis of unknown aetiology (8.2 ± 4.8 to 7.8 ± 4.1; ΔASC −0.4 ± 4.7). Only minimal changes were observed in children with possible encephalitis (8.6 ± 3.9 to 8.3 ± 7.1; ΔASC −0.3 ± 5.8). In contrast, an increase in ASC was observed in children with Lyme disease (4.5 ± 3.4 to 6.2 ± 0.4; ΔASC +1.7 ± 3.2) (Table 3).

When considering selected baseline and outcome characteristics, reductions in ASC were observed in children requiring intensive care unit stay (9.5 ± 3.8 to 8.3 ± 4.9; ΔASC −1.1 ± 4.5). In contrast, ASC slightly increased among immunocompromised children (10.1 ± 5.3 to 10.5 ± 4.6; ΔASC +0.5 ± 5.0) and in children with previous neurological conditions (8.1 ± 3.8 to 8.2 ± 5.4; ΔASC +0.1 ± 5.6) (Table 3).

## Discussion

In this retrospective single-centre cohort study, we examined the clinical spectrum of children with suspected CNS infections in the era of conjugate vaccination and routine multiplex PCR diagnostics, with a particular focus on antibiotic use. We found that empiric antibiotic therapy was frequently initiated, especially in younger children, despite bacterial meningitis being rare. Importantly, LP-related diagnostic information was associated with frequent modifications of antibiotic therapy and an overall reduction in antibiotic spectrum coverage, highlighting the potential of rapid diagnostics to support antimicrobial stewardship in paediatric CNS infections.

The principal finding of this study was that antibiotic management was frequently modified after LP results became available. Among children receiving antibiotic therapy, de-escalation occurred in 42.9%, compared with escalation in 13.6%, while therapy remained unchanged in 43.6%. These direct treatment modifications provide clinically intuitive information on how antibiotic management changed during the diagnostic work-up. The accompanying reduction in ASC provides complementary information by quantifying the change in antimicrobial spectrum breadth. This finding suggests that rapid diagnostic information from CSF analysis can play an important role in guiding antimicrobial stewardship by enabling earlier de-escalation of empiric antibiotic therapy. Similar observations have been reported in previous studies evaluating multiplex ME panels, where rapid molecular diagnostics facilitated earlier adjustment of antimicrobial therapy and helped reduce unnecessary antimicrobial exposure in paediatric patients with suspected CNS infections.^18^ However, previous studies mainly assessed test implementation or availability, whereas all patients in our cohort underwent multiplex PCR testing. Our findings therefore emphasize that antimicrobial changes depend on clinical interpretation and integration of results rather than test availability alone. The observed reduction reflects more targeted, pathogen-directed therapy rather than reduced treatment intensity. Spectrum narrowing in confirmed bacterial meningitis indicated de-escalation from broad empiric regimens. Conversely, spectrum coverage increased in some subgroups, particularly immunocompromised patients, likely owing to persistent diagnostic uncertainty and the greater risk of atypical or opportunistic infections requiring broader coverage.

Despite significant post-LP spectrum reduction, antibiotic use remained frequent. De-escalation in definite viral meningitis suggests more targeted therapy but does not imply immediate discontinuation or fewer treatment days. In selected cases, antibiotics may continue despite a viral PCR result until bacterial CSF cultures remain negative, particularly in neonates with pleocytosis and enteroviral infection. Although this may appear to limit the stewardship impact of CSF diagnostics, it must be interpreted clinically. All children presented with suspected serious infection, often with possible CNS involvement, and LP frequently aimed to exclude CNS infection rather than infection altogether. Thus, clinically relevant infection could remain suspected despite unremarkable CSF findings, making continued antibiotic therapy medically plausible and potentially appropriate.

Our data also suggest that the decision to initiate empiric antibiotic therapy is strongly influenced by patient age, clinical presentation and the underlying diagnostic classification. Antibiotic therapy was initiated particularly frequently in younger children, with very high proportions in neonates and infants, whereas antibiotic use decreased markedly in older age groups. This age-dependent prescribing pattern is consistent with previous paediatric literature, which shows that infants are more likely to receive empiric anti-infective therapy because of non-specific clinical presentations and a higher risk of bacterial meningitis, while older children may more often be managed without empiric treatment due to more specific symptoms.^2,9^ Several clinical features associated with CNS infection appeared to influence treatment decisions. Antibiotics were used more frequently in children with bulging fontanelle, neck stiffness, fever, irritability, rash or prolonged altered consciousness. Use also varied by diagnosis, with the highest proportions in CNS Lyme disease and definite bacterial meningitis and lower rates in febrile convulsions and the control group.

Stratified analyses showed that post-LP antibiotic use remained closely linked to clinical context. Treatment was more often continued when infection was considered likely, whereas greater reductions occurred in conditions with a lower probability of bacterial infection, such as febrile seizures and the control group. This suggests that diagnostic findings were integrated with clinical risk assessment rather than interpreted in isolation. Overall, empiric prescribing was shaped by age-related risk, symptoms suggestive of CNS infection and diagnostic classification.

Several studies show that the mere availability of CSF multiplex PCR testing does not necessarily reduce antibiotic use.^7,9^ Accordingly, some studies found no significant differences in empiric antibiotic therapy between patients with and without the FilmArray^®^ ME panel, and treatment is often continued even after negative results, likely due to limited guidance and integration into clinical practice.^19^ Another study demonstrated that structured clinical integration of rapid diagnostics enables earlier adjustment of antimicrobial therapy, highlighting that the test alone has limited impact.^2^ Instead, its effectiveness depends on appropriate clinical interpretation and integration into decision-making processes, particularly when embedded within antimicrobial stewardship programmes.

The marked post-pandemic increase in children undergoing LP for suspected CNS infection in our cohort—from 9.6 and 9.7 patients per month before and during the pandemic, respectively, to 17.4 patients per month thereafter—is consistent with the broader resurgence of paediatric infectious diseases described by Nygaard et al.^20^ Their review showed that many childhood infections declined during the initial pandemic period and re-emerged, sometimes with unusually high incidence or altered seasonality, after non-pharmaceutical interventions were lifted. In our cohort, the proportion of children receiving any antibiotic therapy decreased from 67.6% before the pandemic to 56.6% during the pandemic and remained nearly unchanged at 56.2% in the post-pandemic period. In contrast, the proportion receiving meningitis-directed antibiotic therapy declined continuously from 58.6% before the pandemic to 51.1% during the pandemic and 43.5% thereafter. Thus, the use of any antibiotic therapy remained broadly stable during and after the pandemic, whereas meningitis-directed antibiotic therapy decreased further in the post-pandemic period.

This study has several limitations. First, its single-centre design may limit generalizability to settings with different populations or clinical practices. Second, the retrospective design entails incomplete data, variable documentation and potential misclassification or selection bias, although predefined diagnostic categories improved consistency. FilmArray ME multiplex PCR results were sometimes available concurrently with routine CSF parameters, making it difficult to attribute treatment decisions specifically to PCR rather than the overall CSF profile. Finally, the study period included the COVID-19 pandemic, during which changes in healthcare-seeking behaviour, pathogen circulation, infection-control measures, case mix and empirical prescribing may have introduced temporal heterogeneity. Although an exploratory period-stratified analysis was performed, the study was not designed to determine a causal pandemic effect on antibiotic use or LP frequency.

A major strength of this study is the inclusion of a large, unselected real-world cohort of children undergoing LP for suspected CNS infection, providing a representative reflection of contemporary clinical practice.

In conclusion, in this large, unselected paediatric cohort, empiric antibiotic therapy was frequently initiated in children with suspected CNS infection. LP-related diagnostic information, including multiplex PCR results, was associated with a significant reduction in ASC, supporting the potential role of integrated CSF diagnostics in antimicrobial stewardship and more targeted therapy.

## Supplementary Material

dlag199_Supplementary_Data

## Data Availability

The data supporting the findings of this study are not publicly available due to ethical restrictions, as individual-level data sharing is not in accordance with the approved ethics committee vote. Aggregated data may be made available upon reasonable request. Access to anonymized data may be granted after review and approval of a research proposal by the responsible ethics committee. Approved data will be shared through a secure online platform.

## Transparency declarations

Yannik Vollmuth reports having received travel support from CSL Behring, outside the submitted work. Johannes Hübner reports having received speaker and/or consultancy fees from GSK, MSD, Sanofi, Pfizer, BioNTech, Moderna, Seqirus, AstraZeneca, bioMérieux and Seegene, all outside the submitted work. Tilmann Schober reports having received speaker and/or consultancy fees from AstraZeneca, Sanofi, bioMérieux and Viatris, all outside the submitted work. Melanie Meyer-Bühn reports having received consultancy fees from bioMérieux and Seegene, all outside the submitted work.

Katrin Baalmann, Uta Behrends, Christoph Klein, Gerhard Dobler, Jana Beer and Benjamin Soric report no conflicts of interest.

## Supplementary data

Figure S1 and Table S1 to S8 are available as Supplementary data at *JAC-AMR* Online.
