# Supplementary material for: Antibiotic use in children with suspected CNS infection in the molecular diagnostic era: a cohort study of 1198 children
Source: JAC Antimicrob Resist. 2026 Sep 2;8(5):dlag199. doi: 10.1093/jacamr/dlag199 (PMC13535220; doi:10.1093/jacamr/dlag199)
Supplement: Supplementary file 1 [file dlag199_supplementary_data.docx]

**Supplementary Material**

Vollmuth et al: Antibiotic Use in Children with Suspected CNS Infection in the Molecular Diagnostic Era: A Cohort Study of 1,198 Children

**Table of Content**

[**Supplementary Methods**	2](#_Toc235972074)

[**Variables of interest and study definitions**	2](#_Toc235972075)

[**Table S1.** STROBE Statement—Checklist	4](#_Toc235972076)

[**Figure S1.** Definition of clinical phenotypes	6](#_Toc235972077)

[**Table S2.** Definition of clinical phenotypes	7](#_Toc235972078)

[**Supplementary Results**	8](#_Toc235972079)

[**Table S3.** Antibiotic treatment according to laboratory parameters	8](#_Toc235972080)

[**Table S4.** Antibiotic treatment according to diagnostic classification	9](#_Toc235972081)

[**Table S5**. Wilcoxon Signed-Rank Test for Antibiotic spectrum coverage (ASC) Change	10](#_Toc235972082)

[**Table S6.** Antibiotic Therapy Changes After LP by Diagnostic Classification	11](#_Toc235972083)

[**Figure S2**. ASC Before and After LP	12](#_Toc235972084)

[**Table S7.** ASC by diagnostic classification (Pre-LP ASC)	13](#_Toc235972085)

[**Table S8.** ASC by diagnostic classification (Post-LP ASC)	14](#_Toc235972086)

[**Table S9.** Antibiotic treatment according to COVID-19 pandemic period	15](#_Toc235972087)

[**Table S10.** ASC Score ^4^	16](#_Toc235972088)

[**References**	18](#_Toc235972089)

# **Supplementary Methods**

### **Variables of interest and study definitions**

Demographic variables included sex and age at presentation. For analytical purposes, age was categorized into six predefined groups: neonates (0–28 days), infants aged 29 days to <6 months, 6–12 months, 1–4 years, 5–9 years, and 10–17 years. Medical history comprised immunocompromised status or immunosuppressive therapy, underlying malignancy, pre-existing neurological conditions and duration of symptoms prior to hospital presentation. Vaccination status was classified as complete, incomplete, or unknown.

Recorded variables at admission included fever and maximum recorded body temperature within ±48 hours of lumbar puncture (LP), headache (assessed only in children aged ≥4 years), neck stiffness, irritability (in children <4 years of age), rash, vomiting, bulging fontanelle (in infants <9 months of age), altered level of consciousness or personality change lasting more than 24 hours, and seizures occurring within 72 hours before or during hospitalization. In addition, newly onset focal neurological deficits occurring within 72 hours before or during hospitalization were recorded. Antimicrobial therapy was documented for all patients. Clinical outcomes included length of hospital stay, admission to the intensive care unit (ICU), initiation of palliative care, and in-hospital mortality. Infections were classified as hospital-acquired if symptom onset occurred more than 48 hours after hospital admission; all remaining cases were considered community-acquired.

Laboratory data included inflammatory and hematologic blood parameters as well as cerebrospinal fluid (CSF) analyses. Blood parameters comprised absolute white blood cell count (WBC) (G/L), absolute neutrophil count (G/L), absolute lymphocyte count (G/L), C-reactive protein (CRP, mg/L), procalcitonin (PCT, ng/mL), and interleukin-6 (IL-6, pg/mL). CSF parameters included corrected WBC count, the proportion of segmented neutrophilic granulocytes (%), lymphocytes (%), and monocytes (%), corrected protein concentration (mg/dL), glucose concentration (mg/dL), and lactate concentration (mmol/L). In cases of traumatic LP predefined correction formulas were applied. CSF leukocyte counts were corrected for erythrocyte contamination using a ratio of 1 × 10⁶/L WBC per 500 × 10⁶/L red blood cells (RBC), and measured protein concentrations were corrected by subtracting 1 mg/dL per 1,000 RBC/µL.

CSF pleocytosis was defined as a CSF WBC count ≥20 × 10⁶/L in neonates aged 0–28 days and >5 × 10⁶/L in children > 28 days, with correction for blood contamination using a ratio of 500 × 10⁶/L RBC to 1 × 10⁶/L WBC ^1^. Definite bacterial meningitis was defined by identification of a relevant bacterial pathogen in CSF, or by CSF pleocytosis with detection of a relevant bacterial pathogen in blood by culture or polymerase chain reaction (PCR), or by a positive CSF gram staining with a corresponding blood pathogen. Relevant pathogens included Neisseria meningitidis, Streptococcus pneumoniae, Haemophilus influenzae, Group B Streptococcus, Escherichia coli, Staphylococcus aureus, and Group A Streptococcus. Definite viral meningitis was defined by CSF pleocytosis with detection of a viral pathogen in CSF or blood, or by detection of enterovirus, parechovirus, or herpes simplex virus (HSV) in CSF irrespective of CSF WBC count. Aseptic meningitis with unknown etiology was defined as CSF pleocytosis without pathogen identification ^1^. (Figure S1 and Table S2).

Encephalitis was classified according to the International Encephalitis Consortium consensus criteria ^2^. The required major criterion was altered mental status, lethargy or personality change lasting ≥24 hours without an alternative cause. Minor criteria included fever ≥38 °C within 72 hours of presentation, seizures not attributable to a preexisting disorder, new-onset focal neurological deficits, CSF WBC count ≥5 cells/mm³, neuroimaging findings suggestive of encephalitis, or electroencephalographic abnormalities consistent with encephalitis. Cases were categorized as possible or probable encephalitis based on the number of fulfilled minor criteria ^2^ (Supplementary Fig 1; Supplementary Table 1). Cases of probable encephalitis, with additional microbiological confirmation of a causative pathogen were defined as confirmed encephalitis^3^.

### **Table S1.** STROBE Statement—Checklist

|  | Item No | Recommendation | Page |
| --- | --- | --- | --- |
| **Title and Abstract** | 1 | (*a*) Indicate the study’s design with a commonly used term in the title or the abstract | 1, 4 |
|  |  | (*b*) Provide in the abstract an informative and balanced summary of what was done and what was found | 4 |
| Introduction | | |  |
| Background/rationale | 2 | Explain the scientific background and rationale for the investigation being reported | 5 |
| Objectives | 3 | State specific objectives, including any prespecified hypotheses | 5 |
| Methods | | |  |
| Study design | 4 | Present key elements of study design early in the paper | 6 |
| Setting | 5 | Describe the setting, locations, and relevant dates, including periods of recruitment, exposure, follow-up, and data collection | 6 |
| Participants | 6 | (*a*) Give the eligibility criteria, and the sources and methods of selection of participants. Describe methods of follow-up | 6, 8,  Fig. 1 |
|  |  | (*b*) For matched studies, give matching criteria and number of exposed and unexposed | n/a |
| Variables | 7 | Clearly define all outcomes, exposures, predictors, potential confounders, and effect modifiers. Give diagnostic criteria, if applicable | 6-7, Suppl. 2-3, 7 |
| Data sources/ measurement | 8* | For each variable of interest, give sources of data and details of methods of assessment (measurement). Describe comparability of assessment methods if there is more than one group | 6, Suppl. 2-3, 7, 15-16 |
| Bias | 9 | Describe any efforts to address potential sources of bias | 12-13 |
| Study size | 10 | Explain how the study size was arrived at | 6, 8  Fig. 1 |
| Quantitative variables | 11 | Explain how quantitative variables were handled in the analyses. If applicable, describe which groupings were chosen and why | 7, Suppl. 2, 13-14 |
| Statistical methods | 12 | (*a*) Describe all statistical methods, including those used to control for confounding | 7,  Suppl.10 |
|  |  | (*b*) Describe any methods used to examine subgroups and interactions | 8-10  Tab. 1-3, Suppl. 9-14 |
|  |  | (*c*) Explain how missing data were addressed | Suppl. 8 |
|  |  | (*d*) If applicable, explain how loss to follow-up was addressed | n/a |
|  |  | (*e*) Describe any sensitivity analyses | n/a |
| Results | | |  |
| Participants | 13* | (a) Report numbers of individuals at each stage of study—eg numbers potentially eligible, examined for eligibility, confirmed eligible, included in the study, completing follow-up, and analysed | 8, Fig. 1 |
|  |  | (b) Give reasons for non-participation at each stage | 8 |
|  |  | (c) Consider use of a flow diagram | Fig. 1 |
| Descriptive data | 14* | (a) Give characteristics of study participants (eg demographic, clinical, social) and information on exposures and potential confounders | Tab. 1  Tab. 2  Suppl. Tab. 3 |
|  |  | (b) Indicate number of participants with missing data for each variable of interest | Suppl. 8 |
|  |  | (c) Summarise follow-up time (eg, average and total amount) | n/a |
| Outcome data | 15* | Report numbers of outcome events or summary measures over time | 8-10  Tab. 1-3 |
| Main results | 16 | (*a*) Give unadjusted estimates and, if applicable, confounder-adjusted estimates and their precision (eg, 95% confidence interval). Make clear which confounders were adjusted for and why they were included | 8-10, Tab. 3 |
|  |  | (*b*) Report category boundaries when continuous variables were categorized | 7, Suppl. 2, 13-14 |
|  |  | (*c*) If relevant, consider translating estimates of relative risk into absolute risk for a meaningful time period | n/a |
| Other analyses | 17 | Report other analyses done—eg analyses of subgroups and interactions, and sensitivity analyses | 8-10, Suppl. 9-14 |
| Discussion | | |  |
| Key results | 18 | Summarise key results with reference to study objectives | 11, 13 |
| Limitations | 19 | Discuss limitations of the study, taking into account sources of potential bias or imprecision. Discuss both direction and magnitude of any potential bias | 12-13 |
| Interpretation | 20 | Give a cautious overall interpretation of results considering objectives, limitations, multiplicity of analyses, results from similar studies, and other relevant evidence | 11-13 |
| Generalisability | 21 | Discuss the generalisability (external validity) of the study results | 12-13 |
| Other information | | |  |
| Funding | 22 | Give the source of funding and the role of the funders for the present study and, if applicable, for the original study on which the present article is based | 14 |

### **Figure S1.** Definition of clinical phenotypes

### **Table S2.** Definition of clinical phenotypes

| Definitions |  | Sources |
| --- | --- | --- |
| CSF pleocytosis | A raised CSF WBC count (CSF pleocytosis) was defined as age 0-28 days CSF WBC ≥20 X10^6^/L, age >28 days CSF WBC >5 X10^6^/L. The CSF WBC count was corrected for RBC count at a ratio of 500X10^6^/L RBC:1 X10^6^/L WBC. | ^1^ |
| Definite bacterial meningitis | Bacterial pathogen in CSF, or raised CSF WBC and relevant pathogen present in blood on culture or PCR, or CSF gram stain positive and corresponding pathogen present in blood. Relevant pathogens in blood included *N. meningitidis, S. pneumoniae, H. influenzae,* Group B *Streptococcus*, *E. coli*, *S. aureus* and Group A *Streptococcus*. | ^1^ |
| Definite viral meningitis | Raised CSF WBC and viral pathogen found in CSF or blood; or enterovirus, parechovirus or HSV present in CSF with a raised or normal CSF WBC. | ^1^ |
| Aseptic meningitis with unknown aetiology | Raised CSF WBC and no causative pathogen identified. | ^1^ |
| Possible encephalitis,  probable encephalitis | Major Criterion (required): Patients presenting to medical attention with altered mental status (defined as decreased or altered level of consciousness, lethargy or personality change) lasting ≥24 h with no alternative cause identified  Minor Criteria (2 required for possible encephalitis, ≥3 required for probable encephalitis):  Documented fever ≥38° C (100.4°F) within the 72 h before or after presentation, generalized or partial seizures not fully attributable to a preexisting seizure disorder, new onset of focal neurologic findings, CSF WBC count ≥5/cubic mm, Abnormality of brain parenchyma on neuroimaging suggestive of encephalitis that is either new from prior studies or appears acute in onset, Abnormality on electroencephalography that is consistent with encephalitis and not attributable to another cause | ^2^ |
| Confirmed encephalitis | Confirmed encephalitis was defined as fulfillment of all criteria for probable encephalitis, with additional microbiological confirmation of a causative pathogen | ^2^ |
| Diagnosis of a complex febrile seizure | All patients who were discharged home with a diagnosis of a complex febrile seizure in their discharge letter. |  |
| CNS Lyme  disease | Patients with neurological symptoms who underwent LP and had serologically confirmed Borrelia infection |  |
| Control Group | All patients without pleocytosis, without pathogen detection in CSF multiplex PCR, and without a diagnosis of possible or probable encephalitis |  |

# **Supplementary Results**

### **Table S3.** Antibiotic treatment according to laboratory parameters

|  | Any Antibiotic Therapy | meningitis-directed antibiotic therapy | Antibiotic Change after LP Results | No antibiotic therapy | Total |
| --- | --- | --- | --- | --- | --- |
| n | 730 | 622 | 412 | 468 | 1198 |
| **Blood Parameters**  (median, IQR) |  |  |  |  |  |
| WBC absolute (G/L) § | 10.3 (7.0-15.8) | 10.8 (7.4-16.1) | 10.0 (6.9-15.9) | 7.7 (6.0-10.4) | 9.0 (6.4-13.3) |
| Absolute neutrophil count (G/L)* | 5.9 (3.1-10.1) | 6.1 (3.3-10.4) | 5.7 (2.9-9.9) | 3.6 (2.6-5.9) | 4.8 (2.8-8.5) |
| Absolute lymphocyte count (G/L)* | 2.8 (1.6-4.3) | 3.0 (1.7-4.5) | 2.8 (1.6-4.4) | 2.4 (1.8-3.6) | 2.6 (1.7-4.0) |
| CRP (mg/L)+ | 1.2 (0.2-4.4) | 1.2 (0.2-4.4) | 0.9 (0.2-3.7) | 0.1 (0.1-0.3) | 4.0 (1.0-27.0) |
| PCT (ng/mL)++ | 0.3 (0.1-2.2) | 0.3 (0.1-2.3) | 0.3 (0.1-1.7) | 0.1 (0.1-0.2) | 0.2 (0.1-1.1 |
| IL-6 (pg/mL)+++ | 64.7 (22.5-238.8) | 72.4 (22.5-289.5) | 56.6 (22.5-178.0) | 8.8 (3.0-29.8) | 46.2 (13.0-168.0) |
| CRP (>8 mg/dl) % | 97 (13.3) | 78 (12.5) | 47 (11.4) | 59 (12.6) | 156 (13.0) |
| PCT (>0.5 ng/mL)% | 139 (19.0) | 116 (18.6) | 68 (16.5) | 18 (3.8) | 157 (13.1) |
| IL-6 (>60 pg/ml) % | 235 (32.2) | 205 (33.0) | 131 (31.8) | 15 (3.2) | 250 (20.9) |
| **CSF Parameters**  (median, IQR) |  |  |  |  |  |
| Pleocytosis | 160 (21.9) | 138 (22.2) | 88 (21.4) | 36 (7.7) | 196 (16.4) |
| Corrected CSF WBC count | 2.0 (0.9-10.6) | 2.0 (1.0-10.9) | 1.6 (0.9-10.2) | 1.0 (0.0-2.0) | 1.0 (0.7-4.0) |
| CSF Segmented neutrophilic granulocytes (%)# | 33.0 (6.0-58.0) | 33.0 (6.0-61.0) | 35.5 (4.0-62.5) | 17.0 (4.0-51.0) | 32.0 (5.0-57.3) |
| CSF Lymphocytes (%)# | 34.0 (16.0-68.0) | 34.0 (15.5-66.0) | 38.5 (16.0-85.3) | 71.0 (36.8-87.3) | 36.0 (17.0-76.0) |
| CSF Monocytes (%)# | 13.0 (6.0-33.0) | 13.0 (6.0-34.0) | 10.0 (4.0-24.0) | 6.5 (4.0-16.3) | 11.0 (5.0-30.0) |
| Corrected CSF Protein (mg/dL) | 36.0 (18.7-67.9) | 38.2 (18.9-69.7) | 24.5 (15.3-51.6) | 21.9 (16.0-29.6) | 26.0 (17.0-52.0) |
| CSF Glucose (mg/dL) | 58.5 (50.5-67.8) | 58.5 (50.6-67.2) | 60.4 (52.2-69.2) | 57.0 (52.9-63.4) | 58.0 (51.7-65.6) |
| CSF Lactate (mmol/L) | 1.6 (1.4-2.0) | 1.6 (1.4-2.0) | 1.5 (1.4-1.8) | 1.4 (1.3-1.7) | 1.51 (1.4-1.8) |

§ For absolute WBC count, data were missing for a total of 42 patients across all categories.

* For leucocyte subgroups, data were missing for a total of 63 patients across all categories.

+ For CRP, data were missing for a total of 62 patients across all categories.

++ For PCT, data were missing for a total of 710 patients across all categories.

+++ For IL-6, data were missing for a total of 638 patients across all categories.

\# These parameters were available only in cases of pleocytosis.

### **Table S4.** Antibiotic treatment according to diagnostic classification

|  | Any Antibiotic Therapy | | meningitis-directed antibiotic therapy | | Antibiotic Change after LP Results | | Total* |
| --- | --- | --- | --- | --- | --- | --- | --- |
|  | Absolute numbers (proportion of any antibiotic therapy (%)) | Proportion of total | Absolute numbers (proportion of antibiotic meningitis therapy (%)) | Proportion of total | Absolute numbers (proportion of antibiotic change after LP (%)) | Proportion of total |  |
| n | 730 | 60.9% | 622 | 51.9% | 412 | 34.4% | 1198 |
| **Diagnostic Groups** | | | | | | | |
| Definite bacterial meningitis | 12 (1.6) | 92.3% | 12 (1.9) | 92.3% | 9 (2.2) | 69.2% | 13 |
| Definite viral meningitis | 71 (9.7) | 88.8% | 60 (9.6) | 75.0% | 47 (11.4) | 58.8% | 80 |
| Aseptic meningitis with unknown etiology | 97 (13.3) | 74.0% | 81 (13.0) | 61.8% | 47 (11.4) | 35.9% | 131 |
| Confirmed/  Probable encephalitis | 44 (6.0) | 81.1% | 40 (6.4) | 75.5% | 21 (5.1) | 39.6% | 53 |
| Possible encephalitis | 27 (3.7) | 79.4% | 23 (3.7) | 67.6% | 15 (3.6) | 44.1% | 34 |
| Diagnosis of febrile convulsions | 88 (12.1) | 66.7% | 77 (12.4) | 58.3% | 54 (13.1) | 40.9% | 132 |
| Lyme Disease | 23 (3.2) | 100.0% | 23 (3.7) | 100.0% | 12 (2.9) | 52.2% | 23 |
| Control group | 490 (67.1) | 54.3% | 414 (66.6) | 45.9% | 273 (66.3) | 30.3% | 902 |

*The total represents the overall study population and is not the sum of the individual diagnostic groups

### **Table S5**. Wilcoxon Signed-Rank Test for Antibiotic spectrum coverage (ASC) Change

| **Wilcoxon Signed Rank Test (ASC Pre-LP/ASC Post-LP)** | |
| --- | --- |
| Total N | 730 |
| Test Statistic | 17110.500 |
| Standard Error | 2338.372 |
| Standardized Test Statistic | -10.874 |
| Asymptotic Sig.(2-sided test) | <.001 |

### **Table S6.** Antibiotic Therapy Changes After LP by Diagnostic Classification

|  | Antibiotic de-escalation | | Antibiotic escalation | | No change in antibiotic therapy | | Total |
| --- | --- | --- | --- | --- | --- | --- | --- |
|  | Absolute numbers (proportion of de-escalation (%)) | Proportion of total | Absolute numbers (proportion of escalation (%)) | Proportion of total | Absolute numbers (proportion of no change (%)) | Proportion of total |  |
| n | 313 | 42.9% | 99 | 13.6% | 318 | 43.6% | 730 |
| **Diagnostic Groups** | | | | | | | |
| Definite bacterial meningitis | 6 (1.9) | 50.0% | 3 (3.0) | 25.0% | 3 (0.9) | 25.0% | 12 |
| Definite viral meningitis | 38 (12.1) | 53.5% | 9 (9.1) | 12.7% | 24 (7.5) | 33.8% | 71 |
| Aseptic meningitis with unknown etiology | 26 (8.3) | 26.8% | 20 (20.2) | 20.6% | 51 (16.0) | 52.6% | 97 |
| Confirmed/  Probable encephalitis | 13 (4.2) | 29.5% | 8 (8.1) | 18.2% | 23 (7.2) | 52.3% | 44 |
| Possible encephalitis | 10 (3.2) | 37.0% | 6 (6.1) | 22.2% | 11 (3.5) | 40.7% | 27 |
| Diagnosis of febrile convulsions | 48 (15.3) | 54.5% | 7 (7.1) | 8.0% | 33 (10.4) | 37.5% | 88 |
| Lyme Disease | 1 (0.3) | 4.3% | 11 (11.1) | 47.8% | 11 (3.5) | 47.8% | 23 |
| Control group | 219 (70.0) | 44.7% | 52 (52.5) | 10.6% | 219 (68.9) | 44.7% | 490 |
| **Selected Baseline and outcome characteristics** | | | | | | | |
| Immunocompromised | 8 (2.6) | 15.4 | 14 (14.1) | 26.9 % | 30 (9.4) | 57.7% | 52 |
| Malignancy | 6 (1.9) | 20.0 | 7 (7.1) | 23.3 % | 17 (5.3) | 56.7% | 30 |
| Previous neurological condition | 13 (4.2) | 26.5 | 11 (11.1) | 22.4 % | 25 (7.9) | 51.0% | 49 |
| ICU stay | 87 (27.8) | 32.6 | 36 (36.4) | 13.5 % | 144 (45.3) | 53.9% | 267 |

### **Figure S2**. ASC Before and After LP

### **Table S7.** ASC by diagnostic classification (Pre-LP ASC)

|  | None (0) | | Moderate (1-9) | | Broad (>10) | | Total |
| --- | --- | --- | --- | --- | --- | --- | --- |
|  | Absolute numbers (proportion of none (%)) | Proportion of total | Absolute numbers (proportion of moderate (%)) | Proportion of total | Absolute numbers (proportion of broad (%)) | Proportion of total |  |
| Total | 51 | 7.0% | 306 | 41.9% | 373 | 51.1% | 730 |
| **Diagnostic Groups** | | | | | |  |  |
| Definite bacterial meningitis | 1 (2.0) | 8.3% | 5 (1.6) | 41.7% | 6 (1.6) | 50.0% | 12 |
| Definite viral meningitis | 7 (13.7) | 9.9% | 19 (6.2) | 26.8% | 45 (12.1) | 63.4% | 71 |
| Aseptic meningitis with unknown etiology | 13 (26.5) | 13.4% | 37 (12.1) | 38.1% | 47 (12.6) | 48.5% | 97 |
| Confirmed/  Probable encephalitis | 3 (5.9) | 6.8% | 28 (9.2) | 63.6% | 13 (3.5) | 29.5% | 44 |
| Possible encephalitis | 0 (0.0) | 0.0% | 17 (5.6) | 63.0% | 10 (2.7%) | 37.0% | 27 |
| Diagnosis of febrile convulsions | 4 (7.8) | 4.5% | 80 (26.1) | 90.9% | 4 (1.1) | 4.5% | 88 |
| Lyme Disease | 7 (13.7) | 30.4% | 15 (4.9) | 65.2% | 1 (0.3) | 4.3% | 23 |
| Control group | 27 (52.9) | 5.5% | 192 (62.7) | 39.2% | 271 (72.7) | 55,3% | 490 |
| **Selected Baseline and outcome characteristics** | | | | | |  |  |
| ICU stay | 9 (17.6) | 3.4% | 93 (30.4) | 35.0% | 164 (44.0) | 61.7% | 266 |
| Immunocompromised | 6 (11.8) | 11.5% | 12 (3.9) | 23.1% | 34 (9.1) | 65.4% | 52 |
| Malignancy | 2 (3.9) | 6.7% | 6 (2.0) | 20.0% | 22 (5.9) | 73.3% | 30 |
| Previous neurological condition | 5 (9.8) | 10.2% | 25 (8.2) | 51.0% | 19 (5.1) | 38.8% | 49 |

### **Table S8.** ASC by diagnostic classification (Post-LP ASC)

|  | None (0) | | Moderate (1-9) | | Broad (>10) | | Total |
| --- | --- | --- | --- | --- | --- | --- | --- |
|  | Absolute numbers (proportion of none (%)) | Proportion of total | Absolute numbers (proportion of moderate (%)) | Proportion of total | Absolute numbers (proportion of broad (%)) | Proportion of total |  |
| Total | 135 | 18.5% | 378 | 51.8% | 217 | 29.7% | 730 |
| **Diagnostic Groups** | | | | | |  |  |
| Definite bacterial meningitis | 1 (0.7) | 8.3% | 8 (2.1) | 66.7% | 3 (1.4) | 25.0% | 12 |
| Definite viral meningitis | 22 (16.1) | 31.0% | 26 (6.9) | 36.6% | 23 (10.6) | 32.4% | 71 |
| Aseptic meningitis with unknown etiology | 4 (3.0) | 4.1% | 62 (16.4) | 63.9% | 31 (14.3) | 32.0% | 97 |
| Confirmed/  Probable encephalitis | 7 (5.1) | 15.9% | 27 (7.1) | 61.4% | 10 (4.6) | 22.7% | 44 |
| Possible encephalitis | 6 (4.4) | 22.2% | 11 (2.9) | 40.7% | 10 (4.6) | 37.0% | 27 |
| Diagnosis of febrile convulsions | 46 (33.6) | 52.3% | 36 (9.5) | 40.9% | 6 (2.8) | 6.8% | 88 |
| Lyme Disease | 0 (0.0) | 0.0% | 23 (6.1) | 100.0% | 0 (0.0) | 0.0% | 23 |
| Control group | 81 (60.0) | 16.5% | 257 (68.0) | 52.4% | 152 (70.0) | 31.0% | 490 |
| **Selected Baseline and outcome characteristics** | | | | | |  |  |
| ICU stay | 31 (23.0) | 11.6% | 108 (28.6) | 40.4% | 128 (59.0) | 47.9% | 267 |
| Immunocompromised | 2 (1.5) | 3.8% | 17 (4.5) | 32.7% | 33 (15.2) | 63.5% | 52 |
| Malignancy | 2 (1.5) | 3.3% | 8 (2.1) | 26.7% | 20 (9.2) | 66.7% | 30 |
| Previous neurological condition | 7 (5.2) | 14.3% | 22 (5.8) | 44.9% | 20 (9.2) | 40.8% | 49 |

### **Table S9.** Antibiotic treatment according to COVID-19 pandemic period

|  | Any Antibiotic Therapy | | Meningitis-directed Antibiotic Therapy | | Antibiotic Change after LP Results | | Total | |
| --- | --- | --- | --- | --- | --- | --- | --- | --- |
| Pandemic Period | Absolute numbers (n, % of total) | Patients/  months | Absolute numbers (n, % of total) | Patients/  months | Absolute numbers (n, % of total) | Patients/  months | Absolute numbers (n, % of total) | Patients/  months |
| Pre-pandemic (1 January 2016 to 10 March 2020) | 328 (67.6) | 6.5 | 284 (58.6) | 5.6 | 218 (44.9) | 4.3 | 485 | 9.6 |
| Pandemic (11 March 2020 to 5 May 2023) | 207 (56.6) | 5.5 | 187 (51.1) | 4.9 | 85 (23.2) | 2.2 | 366 | 9.7 |
| Post-pandemic (6 May 2023 to 31 December 2024) | 195 (56.2) | 9.8 | 151 (43.5) | 7.6 | 109 (31.4) | 5.5 | 347 | 17.4 |
| Total | 730 (60.9) | 6.8 | 622 (51.9) | 5.8 | 412 (34.4) | 3.8 | 1198 | 11.1 |
| p-value (Pearson Chi-square) | <0.001 | | <0.001 | | <0.001 | |  |  |

### **Table S10.** ASC Score ^4^

| **Nr.** | **Antibiotic Class** | **Antibiotic** | **ASC Score** |
| --- | --- | --- | --- |
| 1 | Aminoglycoside | Amikacin / amikacin liposomal | **8** |
| 2 |  | Gentamicin | **9** |
| 3 |  | Plazomicin | **9** |
| 4 |  | Tobramycin | **9** |
| 5 | Beta-lactam / beta-lactamase inhibitor combination | Amoxicillin-clavulanate | **7** |
| 6 |  | Ampicillin-sulbactam | **8** |
| 7 |  | Ceftazidime-avibactam | **8** |
| 8 |  | Ceftolozane-tazobactam | **8** |
| 9 |  | Ticarcillin-clavulanate | **10** |
| 10 |  | Piperacillin-tazobactam | **11** |
| 11 |  | Imipenem-cilastatin-relebactam | **13** |
| 12 |  | Meropenem-vaborbactam | **13** |
| 13 | Carbapenem | Ertapenem | **9** |
| 14 |  | Doripenem | **12** |
| 15 |  | Imipenem-cilastatin | **12** |
| 16 |  | Meropenem | **12** |
| 17 | Cephalosporin | Cefadroxil | **3** |
| 18 |  | Cefazolin | **3** |
| 19 |  | Cefixime | **3** |
| 20 |  | Cephalexin | **3** |
| 21 |  | Cefaclor | **4** |
| 22 |  | Cefdinir | **4** |
| 23 |  | Cefpodoxime | **4** |
| 24 |  | Cefprozil | **4** |
| 25 |  | Cefuroxime | **4** |
| 26 |  | Cefiderocol | **5** |
| 27 |  | Cefotaxime | **6** |
| 28 |  | Cefotetan | **6** |
| 29 |  | Cefoxitin | **6** |
| 30 |  | Ceftazidime | **6** |
| 31 |  | Ceftriaxone | **6** |
| 32 |  | Ceftaroline | **7** |
| 33 |  | Cefepime | **8** |
| 34 | Fluoroquinolone | Ciprofloxacin | **9** |
| 35 |  | Norfloxacin | **9** |
| 36 |  | Ofloxacin | **9** |
| 37 |  | Gemifloxacin | **11** |
| 38 |  | Levofloxacin | **12** |
| 39 |  | Moxifloxacin | **13** |
| 40 |  | Delafloxacin | **14** |
| 41 | Folate pathway inhibitor | Sulfamethoxazole-trimethoprim | **7** |
| 42 | Fosfomycin | Fosfomycin | **8** |
| 43 | Glycopeptide | Dalbavancin | **5** |
| 44 |  | Telavancin | **5** |
| 45 |  | Vancomycin | **5** |
| 46 |  | Oritavancin | **6** |
| 47 | Glycylcycline | Eravacycline | **15** |
| 48 |  | Omadacycline | **15** |
| 49 |  | Tigecycline | **15** |
| 50 | Lincosamide | Clindamycin | **6** |
| 51 | Lipopeptide | Daptomycin | **6** |
| 52 | Macrolide | Erythromycin | **3** |
| 53 |  | Clarithromycin | **5** |
| 54 |  | Azithromycin | **6** |
| 55 | Monobactam | Aztreonam | **4** |
| 56 | Nitrofuran | Nitrofurantoin | **7** |
| 57 | Nitroimidazole | Metronidazole | **2** |
| 58 |  | Tinidazole | **2** |
| 59 | Oxazolidinone | Linezolid | **6** |
| 60 |  | Tedizolid | **6** |
| 61 | Penicillin | Dicloxacillin | **2** |
| 62 |  | Nafcillin | **2** |
| 63 |  | Oxacillin | **2** |
| 64 |  | Penicillin G, V | **3** |
| 65 |  | Amoxicillin | **5** |
| 66 |  | Ampicillin | **5** |
| 67 |  | Piperacillin | **7** |
| 68 | Phenicol | Chloramphenicol | **9** |
| 69 | Pleuromutilin | Lefamulin | **7** |
| 70 | Polymyxin | Colistimethate (colistin) | **6** |
| 71 |  | Polymyxin B | **6** |
| 72 | Rifampin | Rifampin | **5** |
| 73 | Streptogramin | Quinupristin-dalfopristin | **5** |
| 74 | Tetracycline | Doxycycline | **7** |
| 75 |  | Tetracycline | **7** |
| 76 |  | Minocycline | **8** |

# **References**

1.	Martin NG, Defres S, Willis L et al. Paediatric meningitis in the conjugate vaccine era and a novel clinical decision model to predict bacterial aetiology. *J Infect* 2024; **88**: 106145.

2.	Venkatesan A, Tunkel AR, Bloch KC et al. Case definitions, diagnostic algorithms, and priorities in encephalitis: consensus statement of the international encephalitis consortium. *Clin Infect Dis* 2013; **57**: 1114-28.

3.	Vollmuth Y, Soric B, Beer J et al. Pediatric meningoencephalitis in the molecular diagnostic era: epidemiological insights from 1198 suspected cases in Germany between 2016 and 2024. *Infection* 2026.

4.	Kakiuchi S, Livorsi DJ, Perencevich EN et al. Days of Antibiotic Spectrum Coverage: A Novel Metric for Inpatient Antibiotic Consumption. *Clin Infect Dis* 2022; **75**: 567-76.
